# Supplementary material for: New Bicyclic Azalide Macrolides Obtained by Tandem Palladium Catalyzed Allylic Alkylation/Conjugated Addition Reaction
Source: Molecules. 2022 Jan 10;27(2):432. doi: 10.3390/molecules27020432 (PMC8781410; doi:10.3390/molecules27020432)
Supplement: Supplementary file 1 [file molecules-27-00432-s001.zip › molecules-1526724-supplementary.pdf]

Supplementary Information

New Bicyclic Azalide Macrolides Obtained by Tandem Palladium Catalysed Allylic Alkylation/Conjugated Addition Reaction

Sulejman Alihodžić, Hana Čipčić Paljetak, Ana Čikoš and Ivaylo Jivkov Elenkov

Figure S1. ^{1}H spectrum, structure, numbering and assignment of **3** in $CDCl_3$ at 25 °C 2
Figure S2.^{13}C- DEPTq spectrum, structure, numbering and assignment of **3** in $CDCl_3$ at 25 °C 3
Figure S3. ^{1}H spectrum of **8a** in $CDCl_3$ at 25 °C........ 4
Figure S4. ^{13}C- DEPTq spectrum of **8a** in $CDCl_3$ at 25 °C 5
Figure S5. ^{1}H spectrum of **8b** in $CDCl_3$ at 25 °C........ 6
Figure S6. ^{13}C- DEPTq spectrum of **8b** in $CDCl_3$ at 25 °C 7
Figure S7. ^{1}H spectrum, structure, numbering and assignment of **10a** in $CDCl_3$ at 25 °C 8
Figure S8. ^{13}C- DEPTq spectrum, structure, numbering and assignment of **10a** in $CDCl_3$ at 25 °C 9
Figure S9. NOESY spectrum of **10a** in $CDCl_3$ at 25 °C........ 10
Figure S10. ^{1}H spectrum, structure, numbering and assignment of **10b** in $CDCl_3$ at 25 °C........ 11
Figure S11.^{13}C- DEPTq spectrum, structure, numbering and assignment of **10b** in $CDCl_3$ at 25 °C 12
Figure S12. NOESY spectrum of **10b** in $CDCl_3$ at 25 °C 13
Figure S13. ^{1}H spectrum of **14a** in $CDCl_3$ at 25 °C........ 14
Figure S14. ^{1}H spectrum of **14b** in $CDCl_3$ at 25 °C........ 15
Figure S15. ^{1}H spectrum of **15a** in $CDCl_3$ at 25 °C........ 16
Figure S16. ^{13}C-APT spectrum of **15a** in $CDCl_3$ at 25 °C 17
Figure S17. ^{1}H spectrum of **15b** in $CDCl_3$ at 25 °C........ 18
Figure S18. ^{13}C-APT spectrum of **15b** in $CDCl_3$ at 25 °C 19

Table S1. Comparison of $^{3}J_{H,H}$ coupling constants for azithromycin, **3**, **10a** and **10b** in $CDCl_3$ at 25 °C; *overlapped, the largest difference marked in red 20

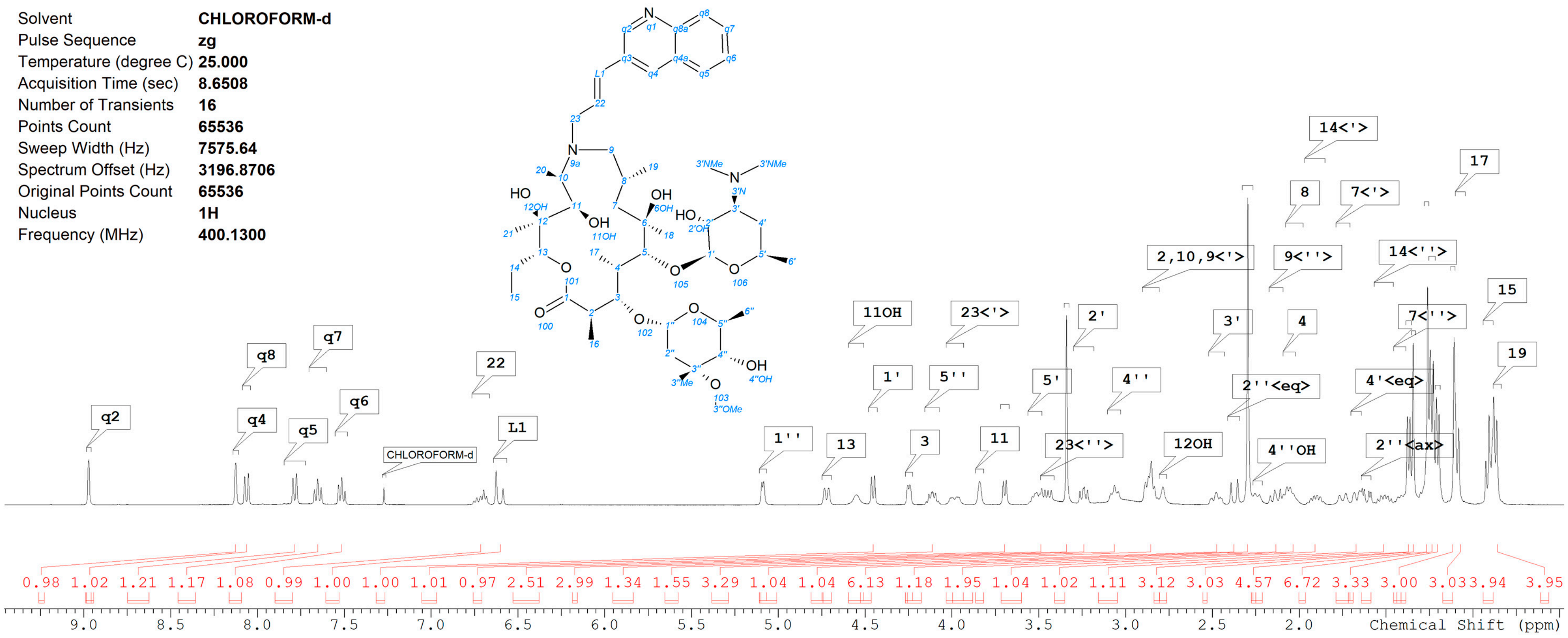

Shift1 (ppm)	H's	Type	J (Hz)	Atom1	Multiplet1
0.872	3.000	br d	7.1	19	M01
0.908	3.000	t	7.4	15	M45
1.092	3.000	d	7.3	17	M02
1.109	3.000	s	-	21	M44
1.203	3.000	d	6.1	6'	M03
1.238	7.000	br d	6.9	4'<ax>, 20, 16	M42
1.261	3.000	s	-	3"Me	M43
1.344	3.000	s	-	18	M04
1.370	3.000	d	6.1	6"	M40
1.422	1.000	dd	15.7, 7.4	7<">	M41
1.510	1.000	ddq	14.6, 9.3, 7.4	14<">	M05
1.613	1.000	dd	15.2, 4.8	2"<ax>	M06
1.668	1.000	br ddd	12.9, 2.0	4'<eq>	M39
1.750	1.000	br d	14.2	7<'>	M07
1.907	1.000	dqd	14.4, 7.7, 1.4	14<'>	M08
2.029	1.000	m	-	8	M46
2.062	1.000	br q	7.4	4	M09
2.140	1.000	dd	11.0, 10.5	9<">	M38
2.243	1.000	br d	10.2	4"OH	M10
2.297	6.000	s	-	3'NMe, 3'NMe	M37
2.375	1.000	d	15.0	2"<eq>	M36
2.479	1.000	ddd	12.9, 9.8, 3.6	3'	M11
2.786	1.000	br s	-	12OH	M12
2.858	3.000	m	-	2, 10, 9<'>	M35
3.064	1.000	br t	8.6	4"	M13
3.242	1.000	br dd	9.8, 7.5	2'	M14
3.341	3.000	s	-	3"OMe	M15
3.457	1.000	br dd	14.4, 7.4	23<">	M16
3.516	1.000	dq	10.4, 6.0	5'	M34
3.697	1.000	d	6.7	5	M17
3.842	1.000	d	1.2	11	M18
3.985	1.000	br dd	13.8, 4.9	23<'>	M19
4.115	1.000	dq	9.6, 6.0	5"	M20
4.249	1.000	br dd	4.0, 1.6	3	M21
4.456	1.000	d	7.2	1'	M22
4.553	1.000	m	-	11OH	M23
4.723	1.000	br dd	9.4, 1.6	13	M24
5.090	1.000	br d	4.6	1"	M25
6.606	1.000	d	16.0	L1	M26
6.718	1.000	ddd	16.0, 7.0, 6.0	22	M27
7.516	1.000	t	7.2	q6	M28
7.654	1.000	t	7.6	q7	M29
7.786	1.000	d	8.2	q5	M30
8.064	1.000	d	8.3	q8	M31
8.126	1.000	s	-	q4	M32
8.972	1.000	s	-	q2	M33

Figure S1. *^{1}H spectrum, structure, numbering and assignment of **3** in $CDCl_3$ at 25 °C*

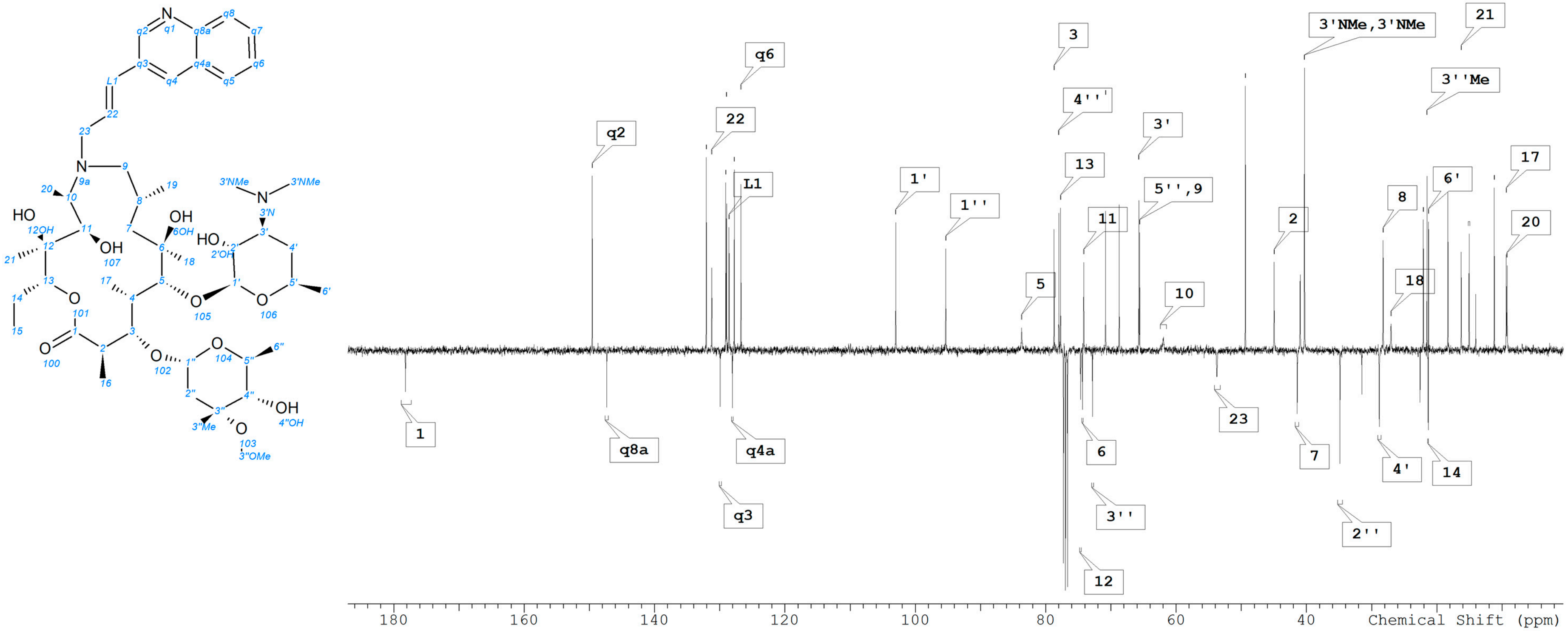

Shift1 (ppm)	C's	Type	Atom1	Multiplet1
9.258	1.000	s	20	M01
9.389	1.000	s	17	M02
11.212	1.000	s	15	M03
15.076	1.000	s	16	M04
16.286	1.000	s	21	M06
18.328	1.000	s	6"	M07
21.317	1.000	s	6'	M08
21.347	1.000	s	14	M48
21.536	1.000	s	3"Me	M09
22.105	1.000	s	19	M10
27.048	1.000	s	18	M11
28.273	1.000	s	8	M12
28.857	1.000	s	4'	M47
34.894	1.000	s	2"	M46
40.333	2.000	s	3'NMe, 3'NMe	M13
40.989	1.000	s		M14
41.441	1.000	s	7	M45
44.985	1.000	s	2	M15
49.403	1.000	s	3"OMe	M16
53.778	1.000	br s	23	M44
61.959	1.000	br s	10	M43
65.619	2.000	s	5", 9	M17
65.736	1.000	s	3'	M18
68.755	1.000	s	5'	M19
70.840	1.000	s	2'	M20
72.838	1.000	s	3"	M42
74.179	1.000	s	11	M21
74.383	1.000	s	6	M41
74.675	1.000	s	12	M25
77.752	1.000	s	13	M22
78.014	1.000	s	4"	M23
78.744	1.000	s	3	M24
83.716	1.000	br s	5	M26
95.353	1.000	s	1"	M27
103.024	1.000	s	1'	M28
126.778	1.000	s	q6	M29
127.799	1.000	s	q5	M30
128.076	1.000	s	q4a	M40
128.601	1.000	s	L1	M31
128.980	1.000	s	q7	M32
129.112	1.000	s	q8	M33
129.957	1.000	s	q3	M39
131.255	1.000	s	22	M34
132.087	1.000	s	q4	M35
147.354	1.000	s	q8a	M38
149.586	1.000	s	q2	M36
178.240	1.000	s	1	M37

Solvent	**CHLOROFORM-d**
Pulse Sequence	**deptqgpsp.2**
Temperature (degree C)	**25.000**
Acquisition Time (sec)	**0.3408**
Number of Transients	**1000**
Points Count	**16384**
Sweep Width (Hz)	**24036.99**
Spectrum Offset (Hz)	**11063.1299**
Original Points Count	**8192**
Nucleus	**13C**
Frequency (MHz)	**100.6128**

Figure S2. *^{13}C- DEPTq spectrum, structure, numbering and assignment of **3** in $CDCl_3$ at 25 °C*

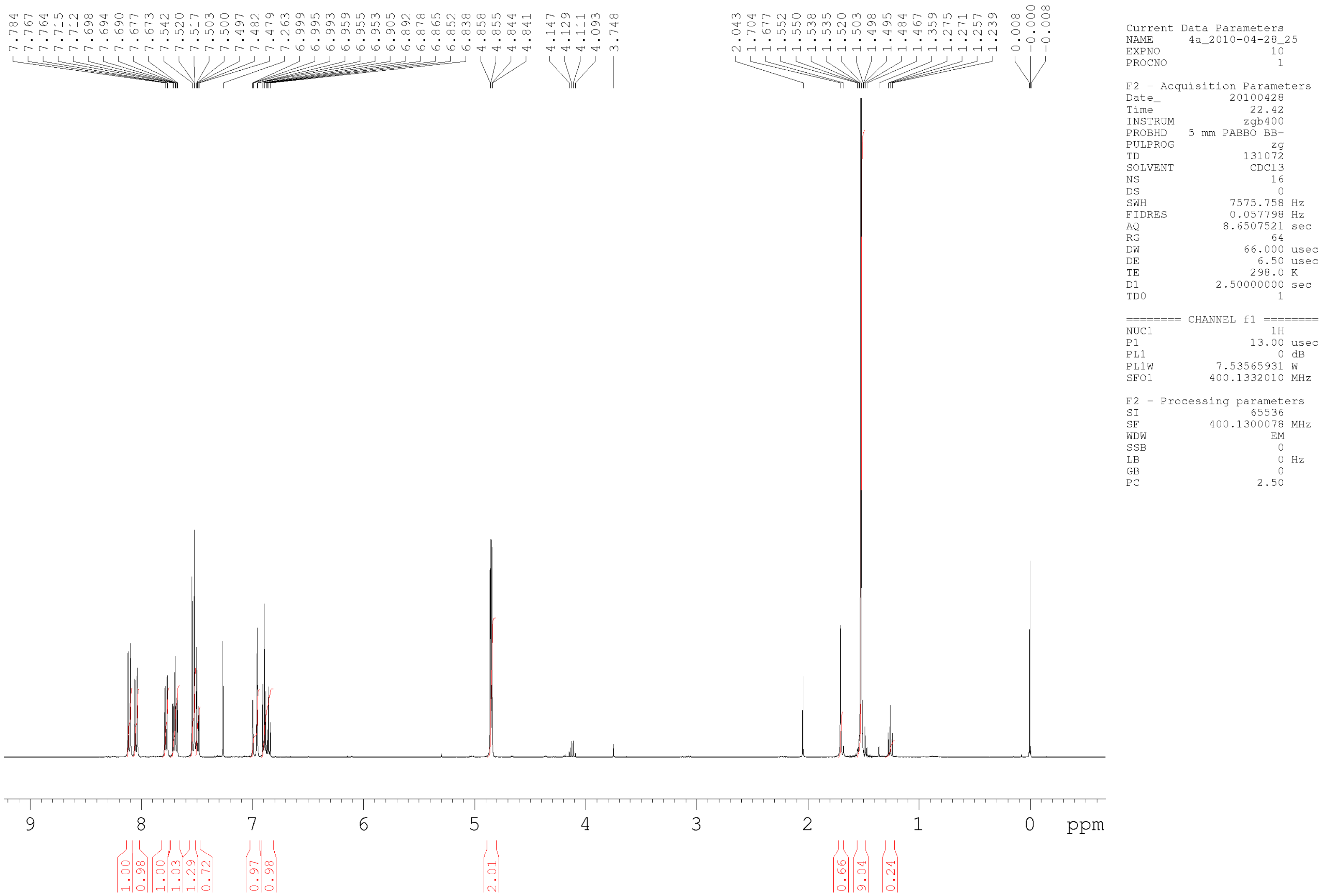

Figure S3. *^{1}H spectrum of* ***8a*** *in $CDCl_3$ at 25 °C*

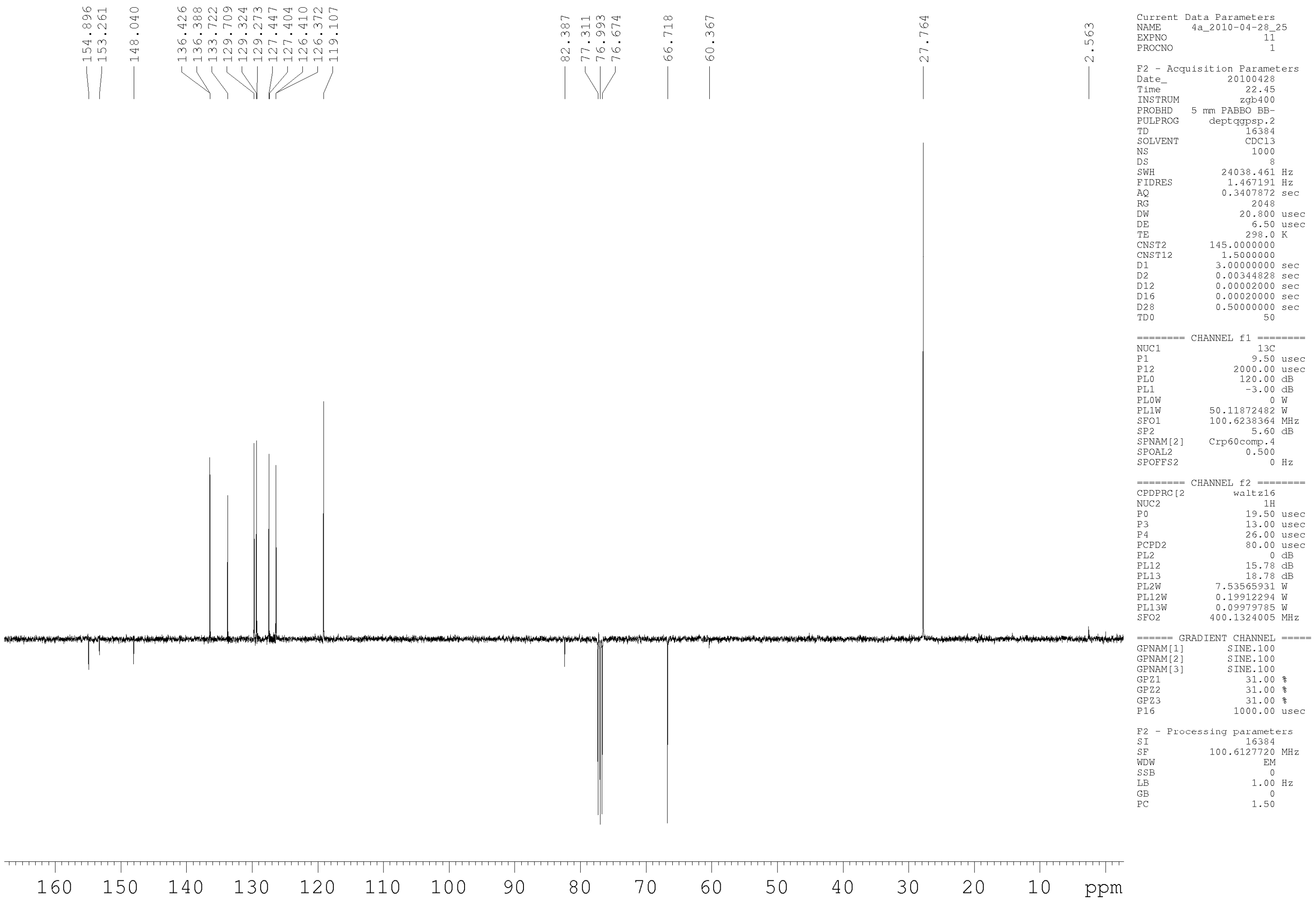

Figure S4. *^{13}C-- DEPTq spectrum of **8a** in $CDCl_3$ at 25 °C*

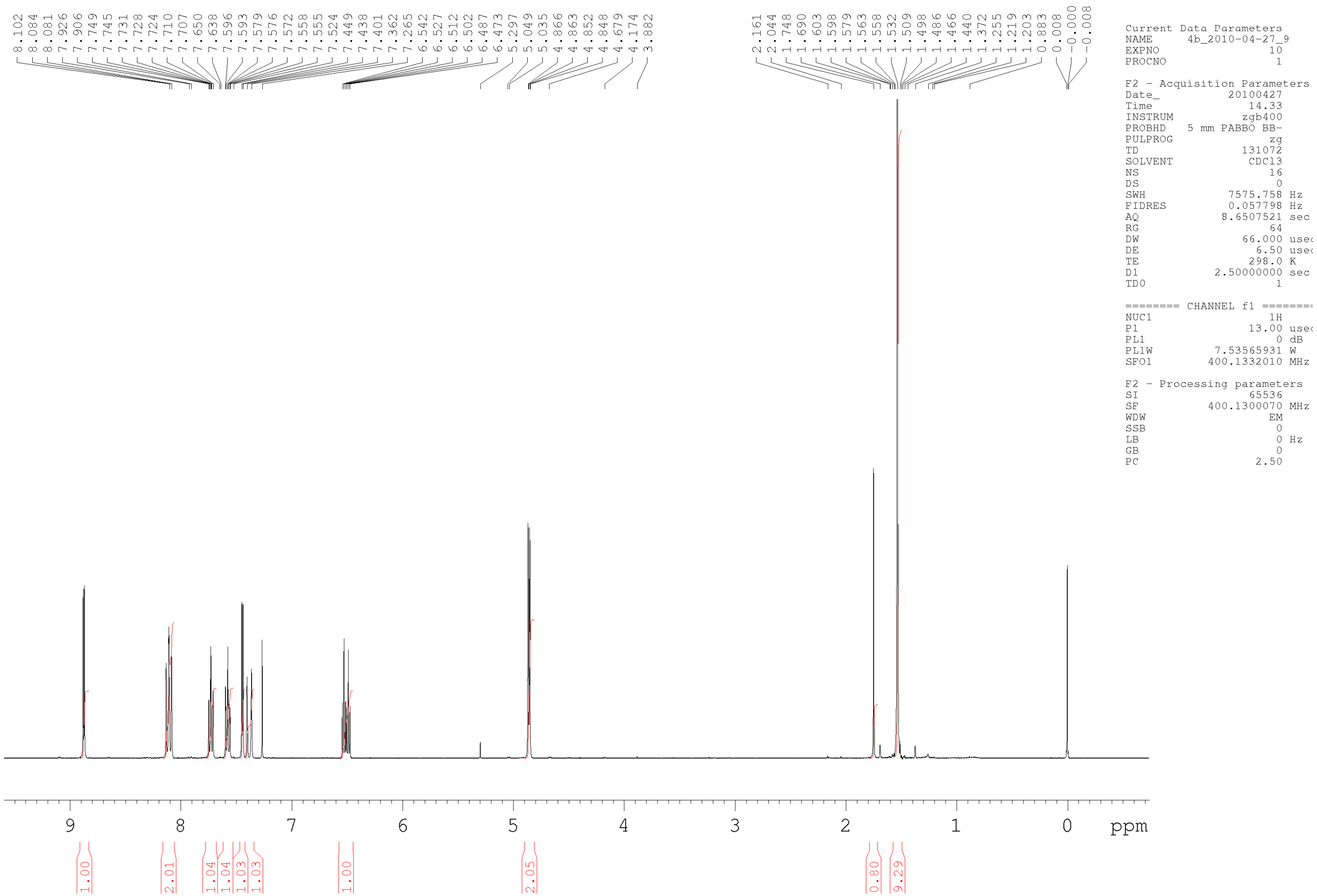

Figure S5. *^{1}H spectrum of **8b** in $CDCl_3$ at 25 °C*

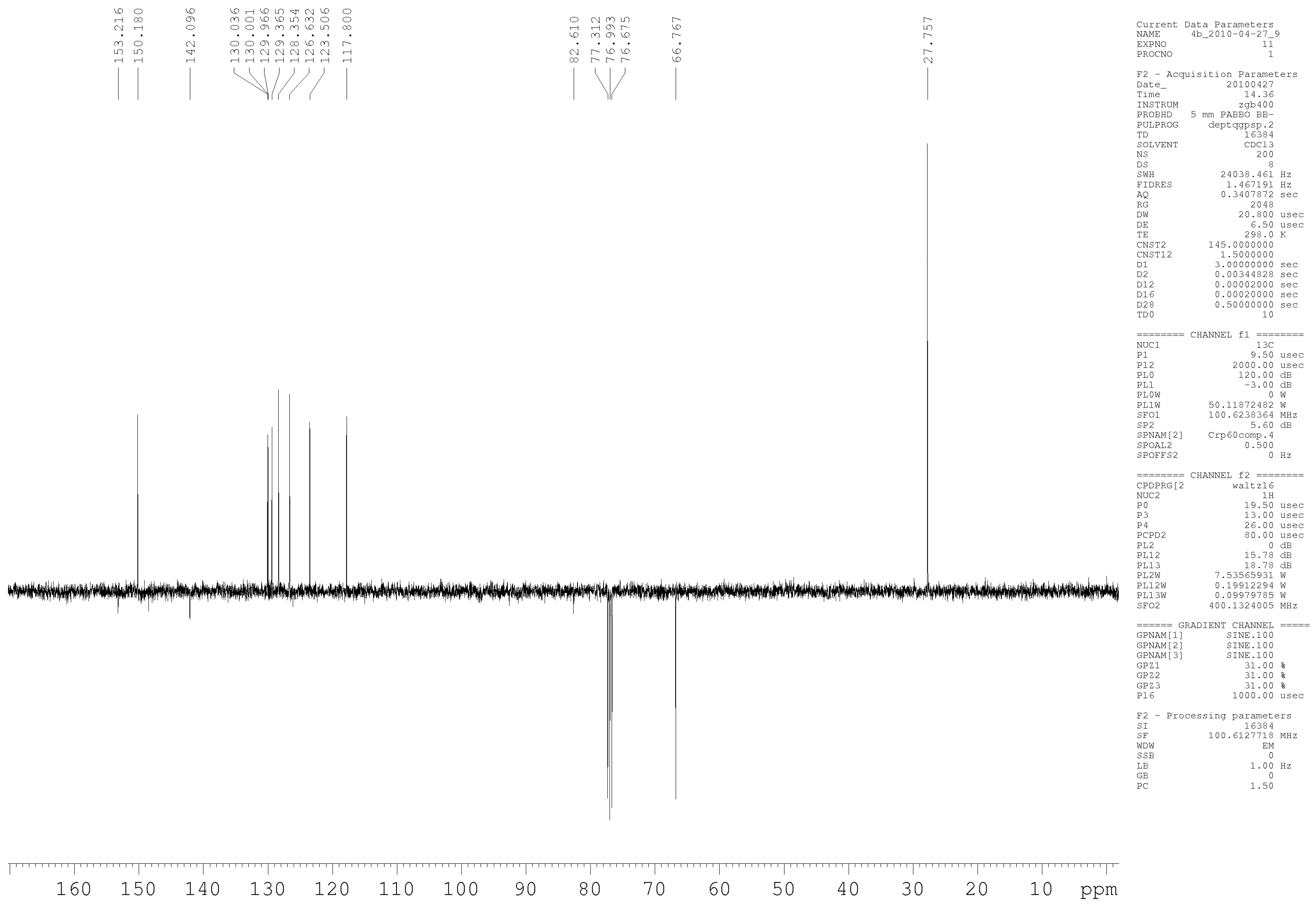

Figure S6. *^{13}C- DEPTq spectrum of **8b** in $CDCl_3$ at 25 °C*

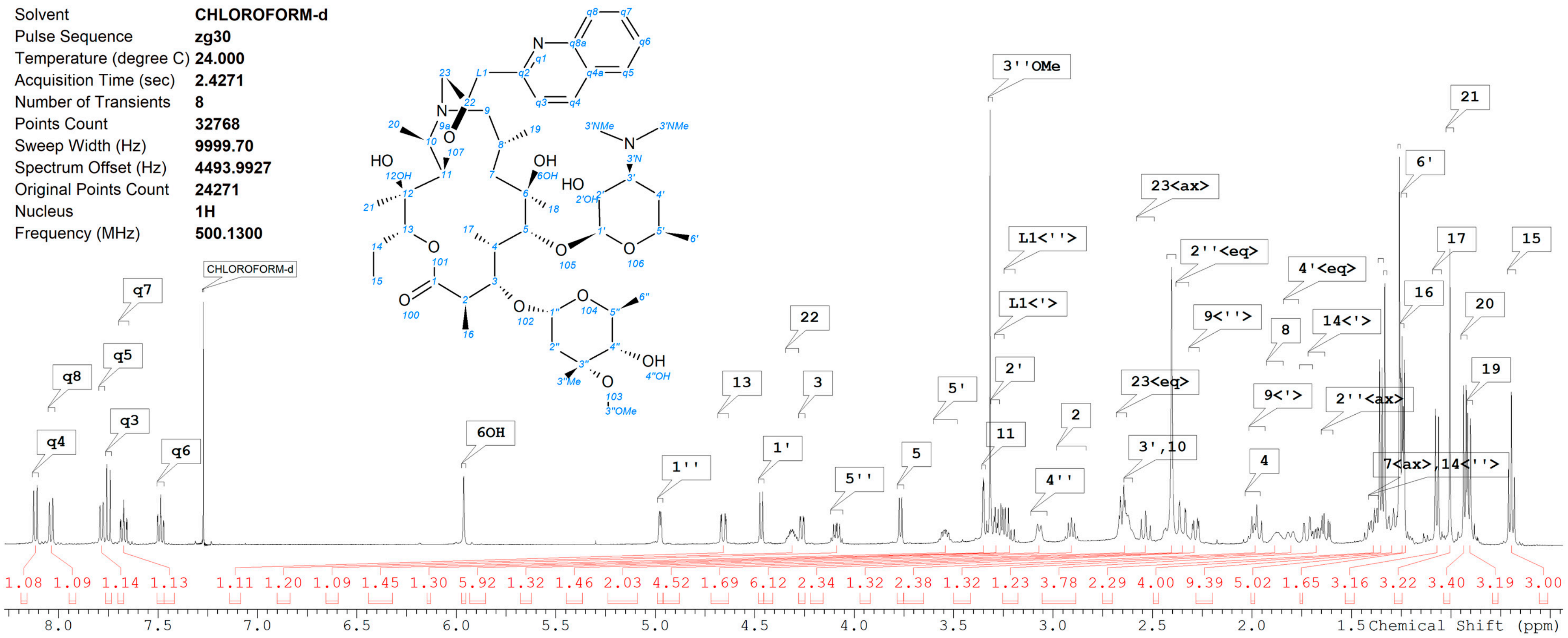

Shift1 (ppm)	H's	Type	J (Hz)	Atom1	Multiplet1	Shift1 (ppm)	H's	Type	J (Hz)	Atom1	Multiplet1	Shift1 (ppm)	H's	Type	J (Hz)	Atom1	Multiplet1
0.696	3.000	t	7.5	15	M01	1.872	1.000	m	-	8	M47	3.543	1.000	dqd	10.2, 6.4, 2.1	5'	M15
0.909	3.000	d	7.0	19	M02	1.977	1.000	br t	12.1	9<'>	M08	3.768	1.000	d	7.0	5	M16
0.930	3.000	d	6.4	20	M38	1.988	1.000	quind	7.5, 1.8	4	M48	4.089	1.000	dq	8.8, 6.2	5"	M17
1.006	3.000	s	-	21	M03	2.284	1.000	dd	12.5, 3.4	9<">	M09	4.264	1.000	dd	8.7, 1.8	3	M18
1.071	3.000	d	7.3	17	M04	2.350	1.000	br dd	14.6, 1.5	2"<eq>	M36	4.315	1.000	br dddd	10.7, 8.2, 4.9, 2.9	22	M29
1.240	3.000	br d	6.1	6'	M05	2.404	6.000	s	-	3'NMe, 3'NMe	M35	4.468	1.000	d	7.3	1'	M19
1.248	3.000	br d	7.3	16	M39	2.535	1.000	dd	11.6, 10.7	23<ax>	M10	4.659	1.000	dd	11.1, 2.0	13	M20
1.260	3.000	s	-	3"Me	M40	2.624	2.000	m	-	3', 10	M11	4.976	1.000	dd	4.6, 1.4	1"	M21
1.301	1.000	td	11.3, 10.2	4'<ax>	M43	2.654	1.000	dd	11.6, 3.1	23<eq>	M34	5.964	1.000	s	-	6OH	M22
1.333	3.000	s	-	18	M06	2.909	1.000	dq	8.7, 7.2	2	M12	7.487	1.000	ddd	8.1, 7.0, 1.1	q6	M23
1.353	3.000	d	6.1	6"	M41	3.070	1.000	br d	8.5	4"	M13	7.673	1.000	ddd	8.4, 6.9, 1.5	q7	M24
1.389	2.000	m	-	7<ax>, 14<">	M42	3.219	1.000	dd	15.0, 8.2	L1<">	M14	7.750	1.000	d	8.5	q3	M25
1.628	1.000	dd	15.0, 4.9	2"<ax>	M07	3.273	1.000	dd	14.0, 4.9	L1<'>	M31	7.784	1.000	dd	8.2, 0.9	q5	M28
1.674	1.000	dqd	14.4, 7.5, 1.8	14<'>	M45	3.295	1.000	dd	10.4, 7.6	2'	M33	8.039	1.000	d	8.2	q8	M26
1.726	1.000	d	14.6	7<eq>	M37	3.317	3.000	s	-	3"OMe	M32	8.117	1.000	d	8.5	q4	M27
1.807	1.000	ddd	12.5, 4.0, 2.1	4'<eq>	M44	3.351	1.000	d	1.8	11	M30						

Figure S7. *^{1}H spectrum, structure, numbering and assignment of **10a** in $CDCl_3$ at 25 °C*

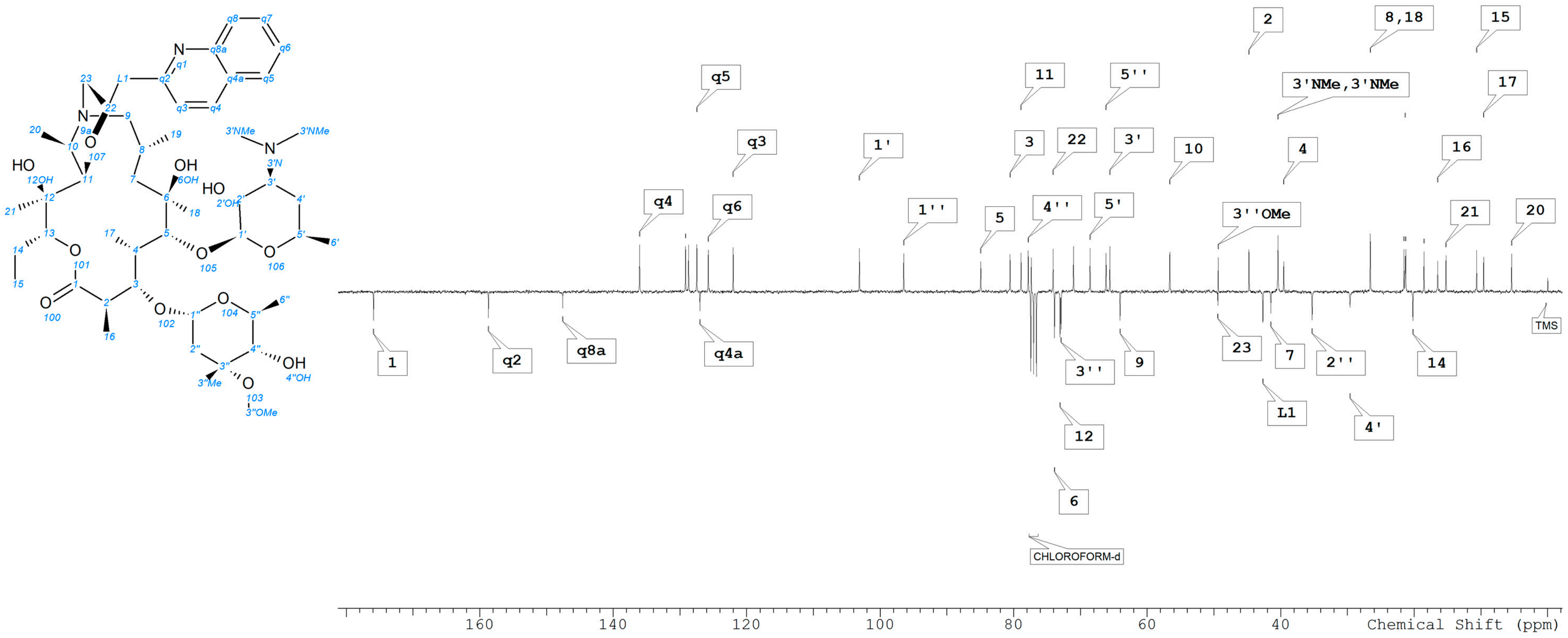

Shift1 (ppm)	C's	Type	Atom1	Multiplet1
5.421	1.000	s	20	M02
9.607	1.000	s	17	M03
10.662	1.000	s	15	M04
15.253	1.000	s	21	M05
16.498	1.000	s	16	M06
18.551	1.000	s	6"	M07
20.201	1.000	s	14	M08
21.279	1.000	s	6'	M09
21.350	1.000	s	19	M10
21.548	1.000	s	3"Me	M11
26.591	2.000	s	8, 18	M12
29.636	1.000	s	4'	M13
35.313	1.000	s	2"	M14
39.563	1.000	s	4	M15
40.427	2.000	s	3'NMe, 3'NMe	M16

Shift1 (ppm)	C's	Type	Atom1	Multiplet1
41.490	1.000	s	7	M17
42.687	1.000	s	L1	M18
44.773	1.000	s	2	M19
49.379	1.000	s	3"OMe	M20
49.419	1.000	s	23	M21
56.634	1.000	s	10	M22
64.080	1.000	s	9	M23
65.618	1.000	s	3'	M24
66.189	1.000	s	5"	M25
68.583	1.000	s	5'	M26
71.065	1.000	s	2'	M27
72.920	1.000	s	3"	M28
73.079	1.000	s	12	M29
73.904	1.000	s	6	M30
74.118	1.000	s	22	M31

Shift1 (ppm)	C's	Type	Atom1	Multiplet1
77.392	1.000	s	13	M48
77.844	1.000	s	4"	M32
78.923	1.000	s	11	M33
80.564	1.000	s	3	M34
84.949	1.000	s	5	M35
96.501	1.000	s	1"	M36
103.146	1.000	s	1'	M37
122.057	1.000	s	q3	M38
125.783	1.000	s	q6	M39
127.036	1.000	s	q4a	M40
127.496	1.000	s	q5	M41
128.733	1.000	s	q8	M42
129.193	1.000	s	q7	M43
136.075	1.000	s	q4	M44
147.596	1.000	s	q8a	M45

Shift1 (ppm)	C's	Type	Atom1	Multiplet1
158.728	1.000	s	q2	M46
175.934	1.000	s	1	M47

Solvent	**CHLOROFORM-d**
Pulse Sequence	**jmod**
Temperature (degree C)	**26.160**
Acquisition Time (sec)	**1.6712**
Number of Transients	**2425**
Points Count	**32768**
Sweep Width (Hz)	**19607.25**
Spectrum Offset (Hz)	**9027.3271**
Original Points Count	**32768**
Nucleus	**13C**
Frequency (MHz)	**75.4678**

Figure S8. *^{13}C- DEPTq spectrum, structure, numbering and assignment of* ***10a*** *in $CDCl_3$ at 25 °C*

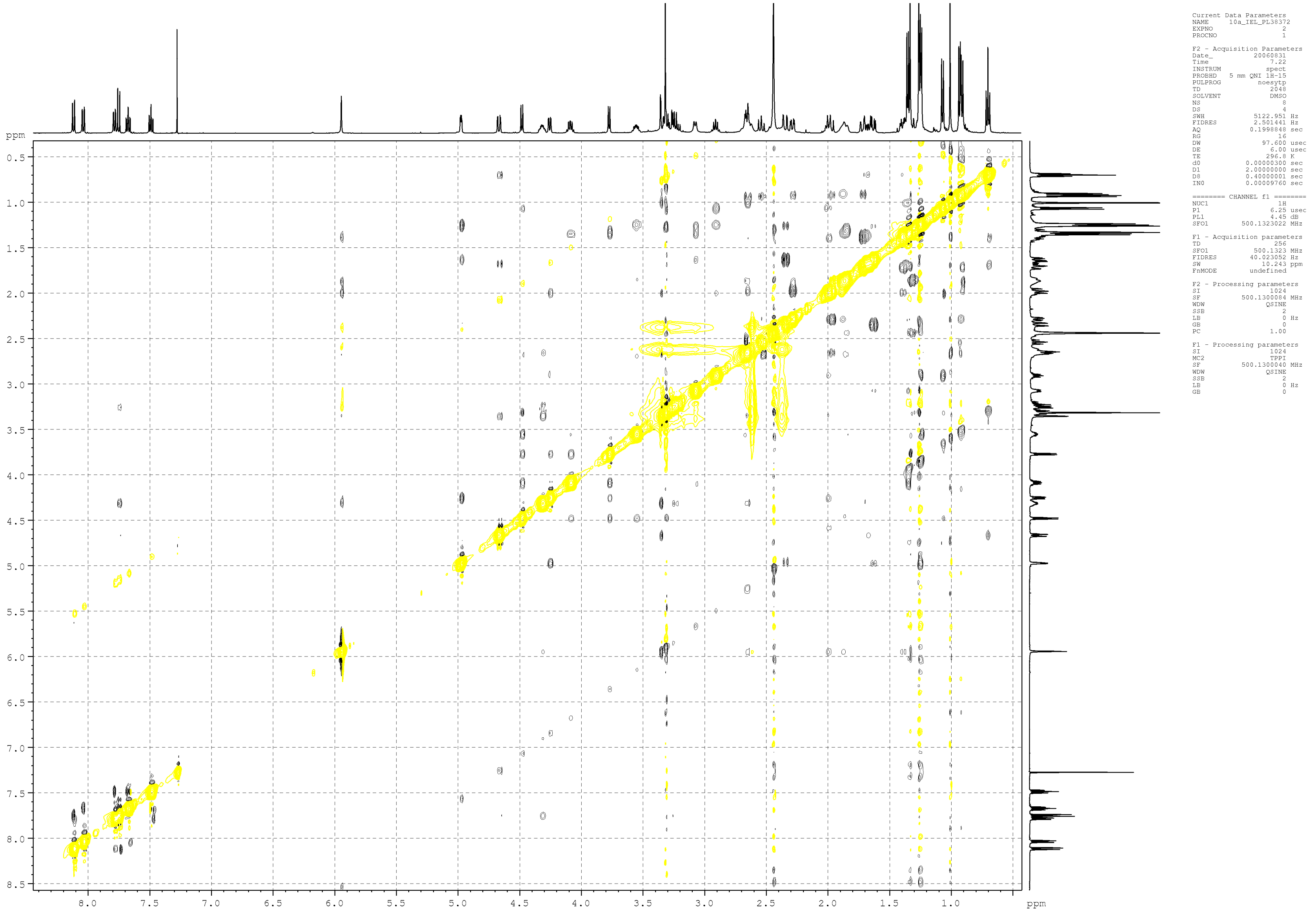

Figure S9. *NOESY spectrum of **10a** in $CDCl_3$ at 25 °C*

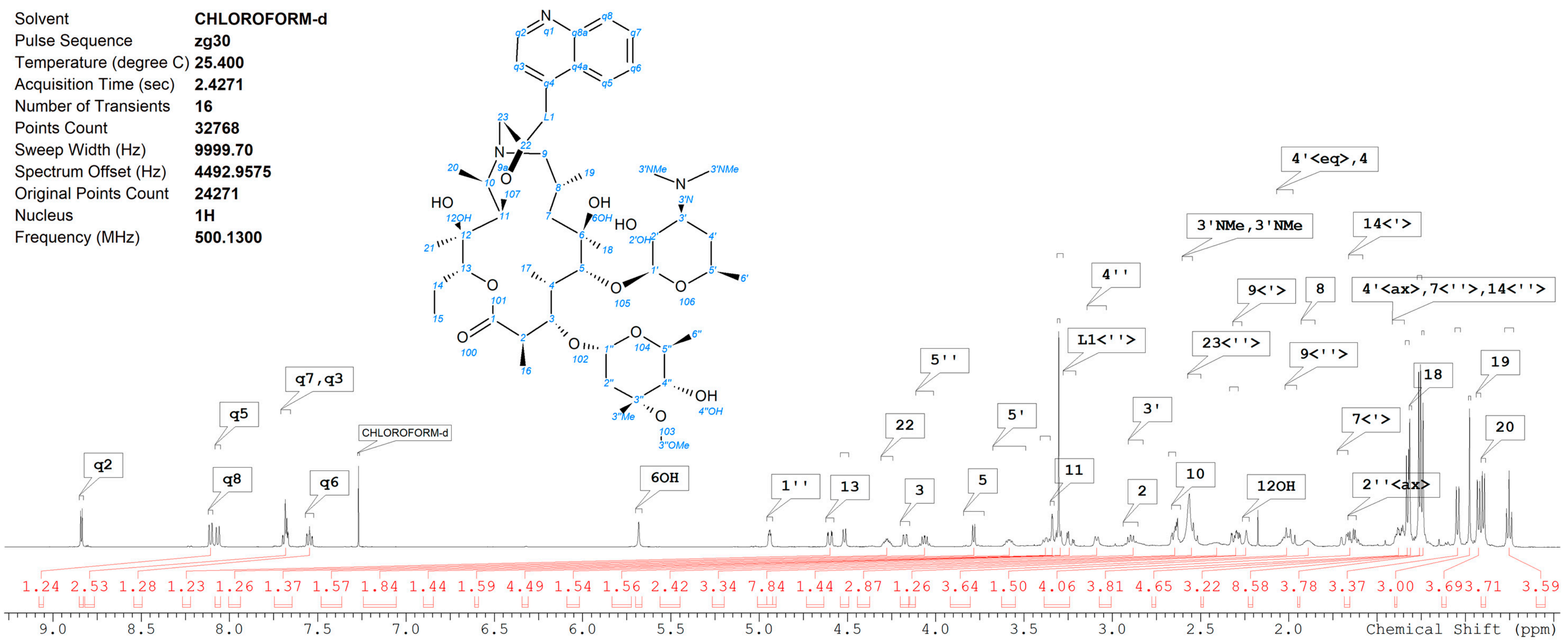

Shift1 (ppm)	H's	Type	J (Hz)	Atom1	Multiplet1
0.753	3.000	t	7.3	15	M01
0.899	3.000	br d	6.4	20	M02
0.927	3.000	br d	7.0	19	M34
0.978	3.000	s	-	21	M03
1.045	3.000	d	7.3	17	M04
1.241	3.000	s	-	3"Me	M06
1.260	6.000	d	5.2	16, 6'	M47
1.316	3.000	s	-	18	M08
1.329	3.000	d	6.4	6"	M46
1.373	3.000	m	-	4'<ax>, 7<">, 14<">	M07
1.630	1.000	dqd	14.4, 7.3, 1.8	14<'>	M45
1.645	1.000	dd	14.8, 4.7	2"<ax>	M44
1.688	1.000	br dd	14.8, 1.1	7<'>	M09
1.891	1.000	m	-	8	M10
1.991	1.000	t	12.5	9<">	M11
2.026	2.000	m	-	4'<eq>, 4	M43
2.242	1.000	br s	-	12OH	M13
2.292	1.000	br dd	12.4, 3.5	9<'>	M42
2.310	1.000	br dd	14.6, 2.4	2"<eq>	M12
2.410	1.000	m	-		M14
2.542	1.000	t	11.6	23<">	M40
2.565	6.000	br s	-	3'NMe, 3'NMe	M41
2.644	1.000	br s	-	10	M16
2.656	1.000	dd	8.5, 3.1	23<'>	M15
2.842	1.000	br s	-	3'	M17
2.897	1.000	dq	9.2, 7.3	2	M39
3.089	1.000	br d	7.9	4"	M18
3.237	1.000	dd	15.6, 4.0	L1<">	M19
3.304	3.000	s	-	3"OMe	M38
3.305	1.000	d	15.0	L1<'>	M37
3.341	1.000	d	1.8	11	M36
3.375	1.000	br dd	9.9, 7.5	2'	M35
3.585	1.000	dqd	10.2, 6.1, 2.0	5'	M20
3.786	1.000	d	6.1	5	M21
4.064	1.000	dq	8.8, 6.4	5"	M22
4.175	1.000	dd	9.0, 1.7	3	M23
4.278	1.000	ddt	10.8, 7.2, 3.7	22	M24
4.518	1.000	d	7.3	1'	M25
4.601	1.000	dd	11.1, 2.0	13	M26
4.941	1.000	dd	4.3, 2.4	1"	M27
5.683	1.000	s	-	6OH	M28
7.548	1.000	ddd	8.2, 7.0, 1.2	q6	M29
7.682	2.000	m	-	q7, q3	M30
8.068	1.000	d	8.2	q5	M31
8.109	1.000	d	7.6	q8	M32
8.840	1.000	d	4.6	q2	M33

Figure S10. *^{1}H spectrum, structure, numbering and assignment of **10b** in $CDCl_3$ at 25 °C*

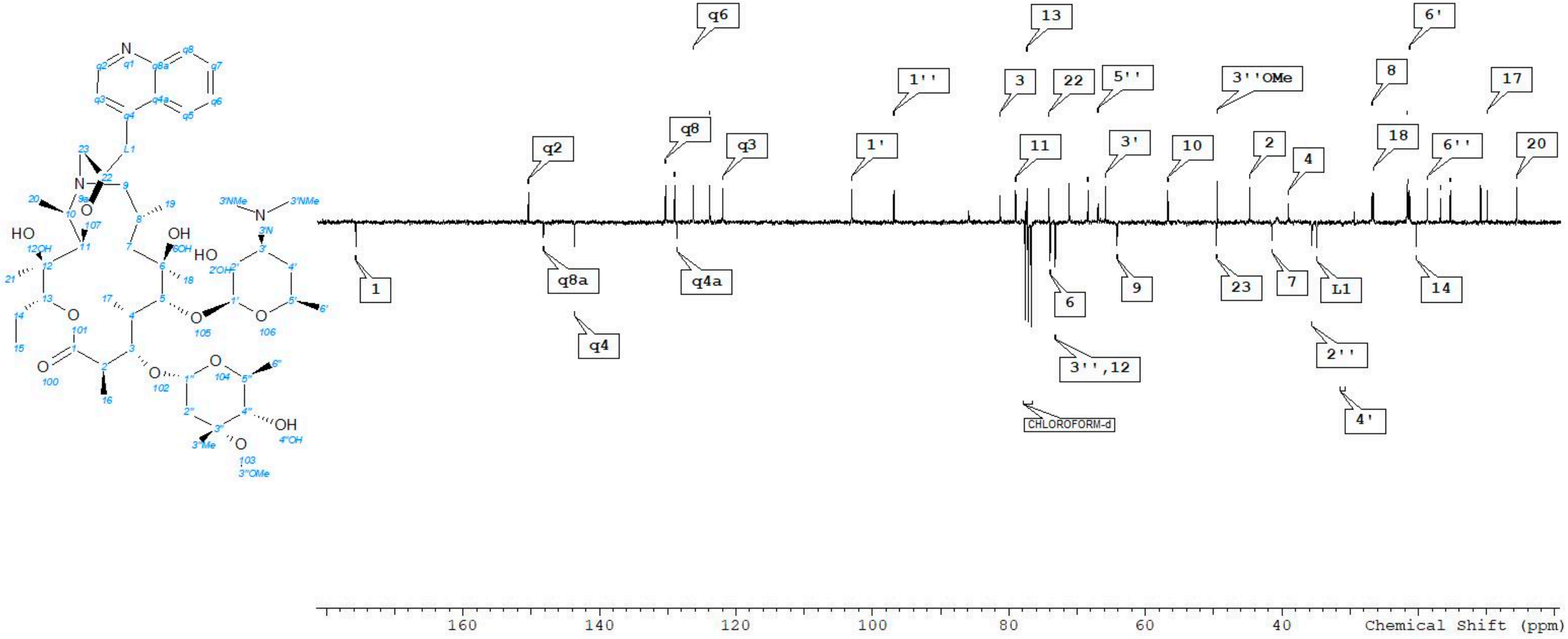

Shift1 (ppm)	C's	Type	Atom1	Multiplet1
5.576	1.000	s	20	M01
9.905	1.000	s	17	M02
10.873	1.000	s	15	M03
15.289	1.000	s	21	M04
16.764	1.000	s	16	M05
18.651	1.000	s	6"	M06
20.300	1.000	s	14	M07
21.252	1.000	s	6'	M08
21.482	1.000	s	19	M09
21.648	1.000	s	3"Me	M10
26.572	1.000	s	18	M11
26.762	1.000	s	8	M12
31.145	1.000	m	4'	M13
34.890	1.000	s	L1	M14
35.579	1.000	s	2"	M15
39.013	1.000	s	4	M16
40.722	2.000	s	3'NMe, 3'NMe	M18
41.439	1.000	s	7	M19
44.714	1.000	s	2	M20
49.439	1.000	s	3"OMe	M21
49.574	1.000	s	23	M22
56.694	1.000	s	10	M23
64.132	1.000	s	9	M24
65.836	1.000	s	3'	M25
66.899	1.000	s	5"	M26
68.413	1.000	s	5'	M27
71.173	1.000	s	2'	M28
73.209	1.000	s		M29
73.209	2.000	s	3", 12	M30
73.924	1.000	s	6	M31
74.106	1.000	s	22	M32
77.333	1.000	s	13	M48
79.030	1.000	s	11	M33
81.258	1.000	s	3	M34
85.873	1.000	s		M35
96.823	1.000	s	1"	M36
103.023	1.000	s	1'	M37
121.974	1.000	s	q3	M38
123.837	1.000	s	q5	M39
126.240	1.000	s	q6	M40
128.642	1.000	s	q4a	M41
128.959	1.000	s	q7	M42
130.299	1.000	s	q8	M43
143.668	1.000	s	q4	M44
148.179	1.000	s	q8a	M45
150.383	1.000	s	q2	M46
175.677	1.000	s	1	M47

Solvent	CHLOROFORM-d
Pulse Sequence	jmod
Temperature (degree C)	25.160
Acquisition Time (sec)	1.6712
Number of Transients	2068
Points Count	32768
Sweep Width (Hz)	19607.25
Spectrum Offset (Hz)	9037.8359
Original Points Count	32768
Nucleus	13C
Frequency (MHz)	75.4677

Figure S11. *^{13}C- DEPTq spectrum, structure, numbering and assignment of **10b** in $CDCl_3$ at 25 °C*

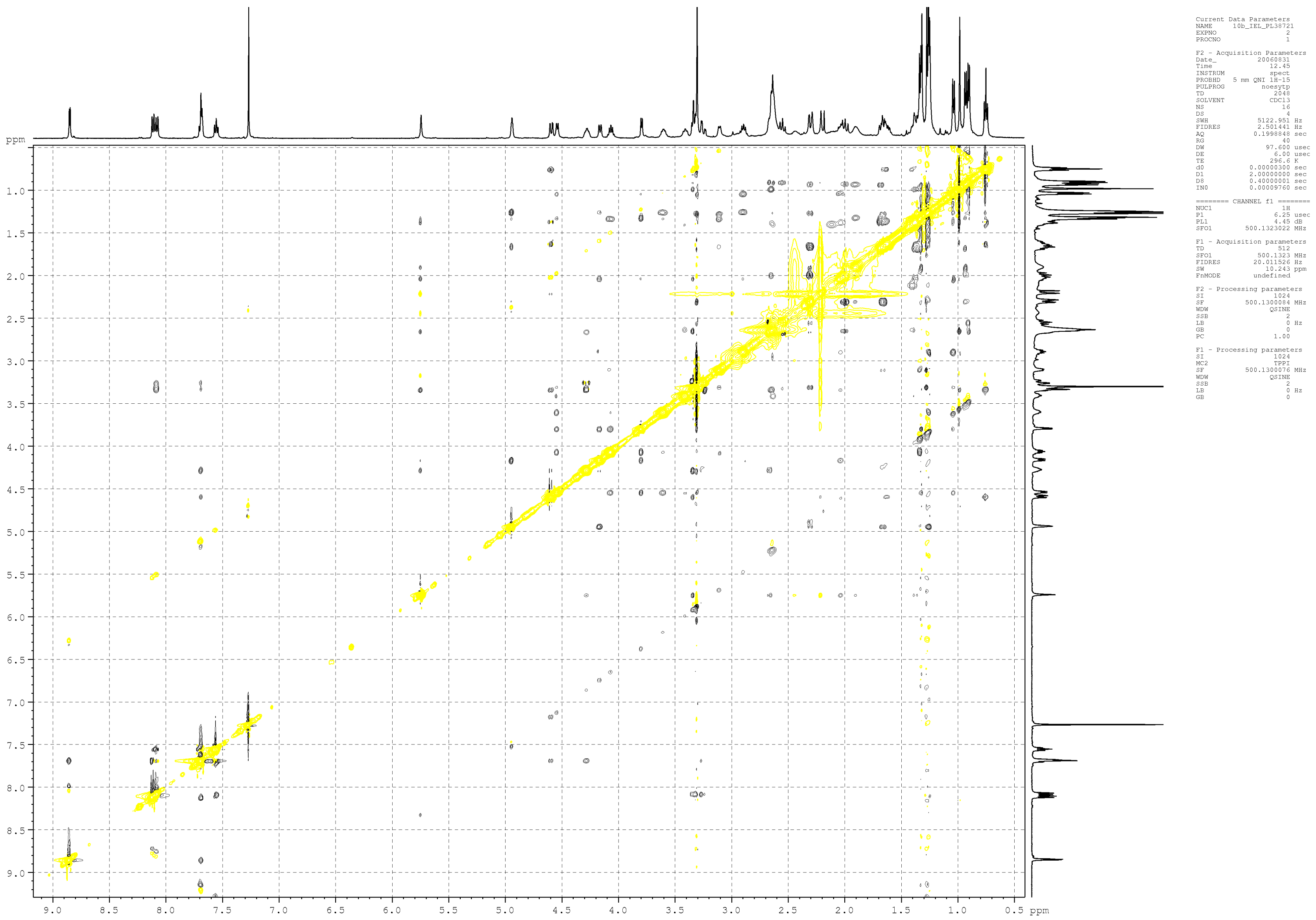

Figure S12. *NOESY spectrum of* ***10b*** *in $CDCl_3$ at 25 °C*

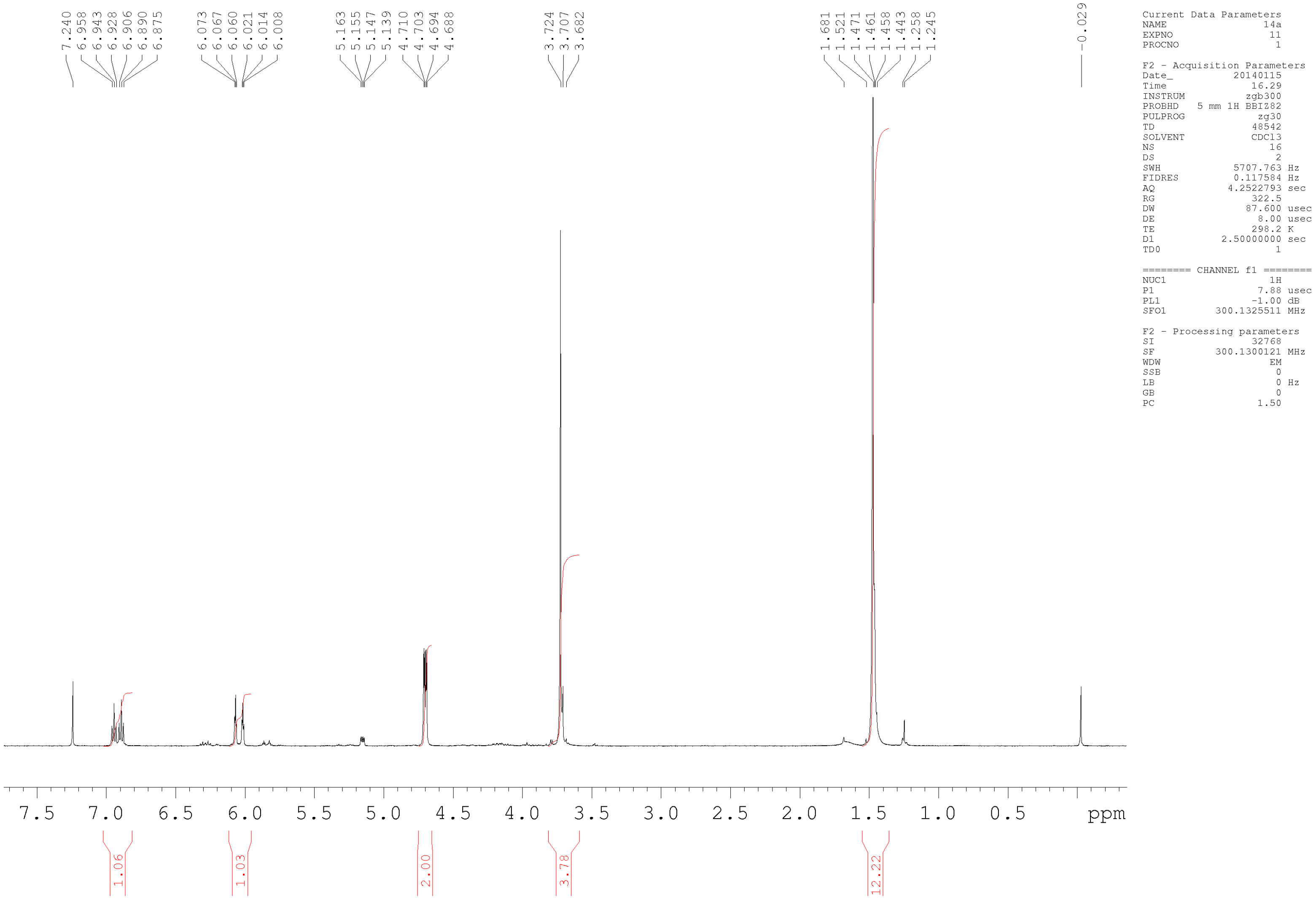

Figure S13. *^{1}H spectrum of **14a** in $CDCl_3$ at 25 °C*

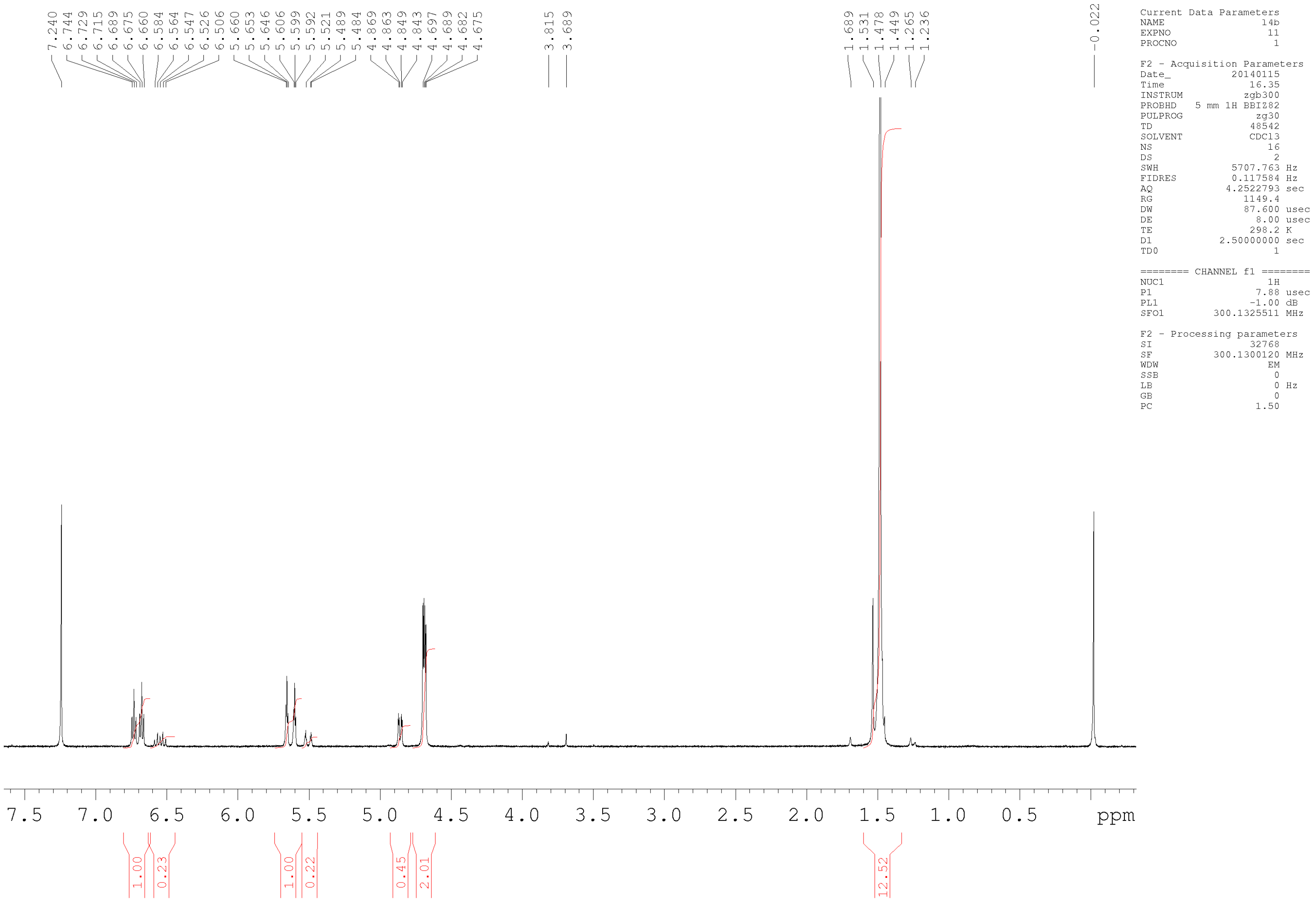

Figure S14. *^{1}H spectrum of* ***14b*** *in $CDCl_3$ at 25 °C*

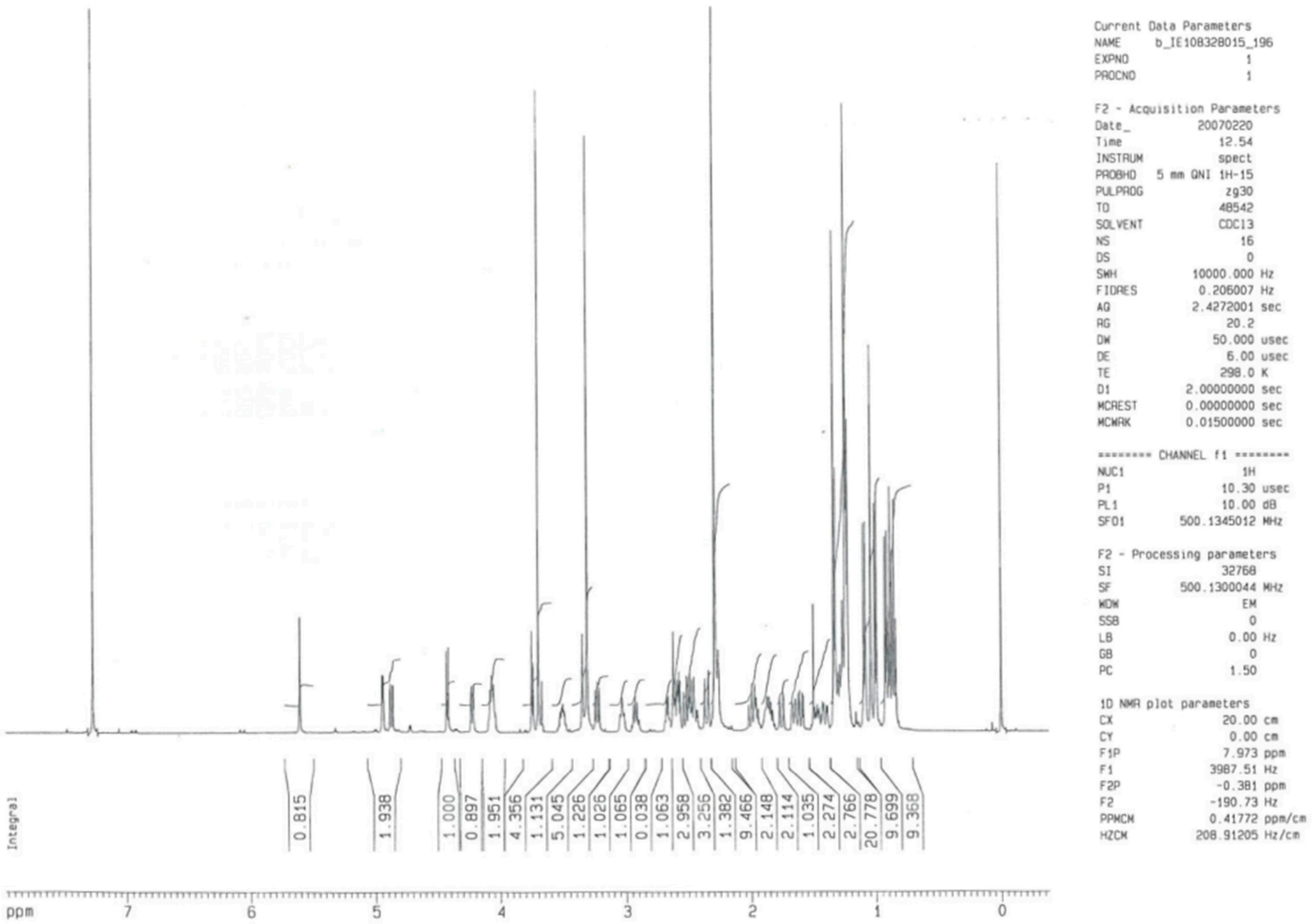

Figure S15. *^{1}H spectrum of **15a** in $CDCl_3$ at 25 °C*

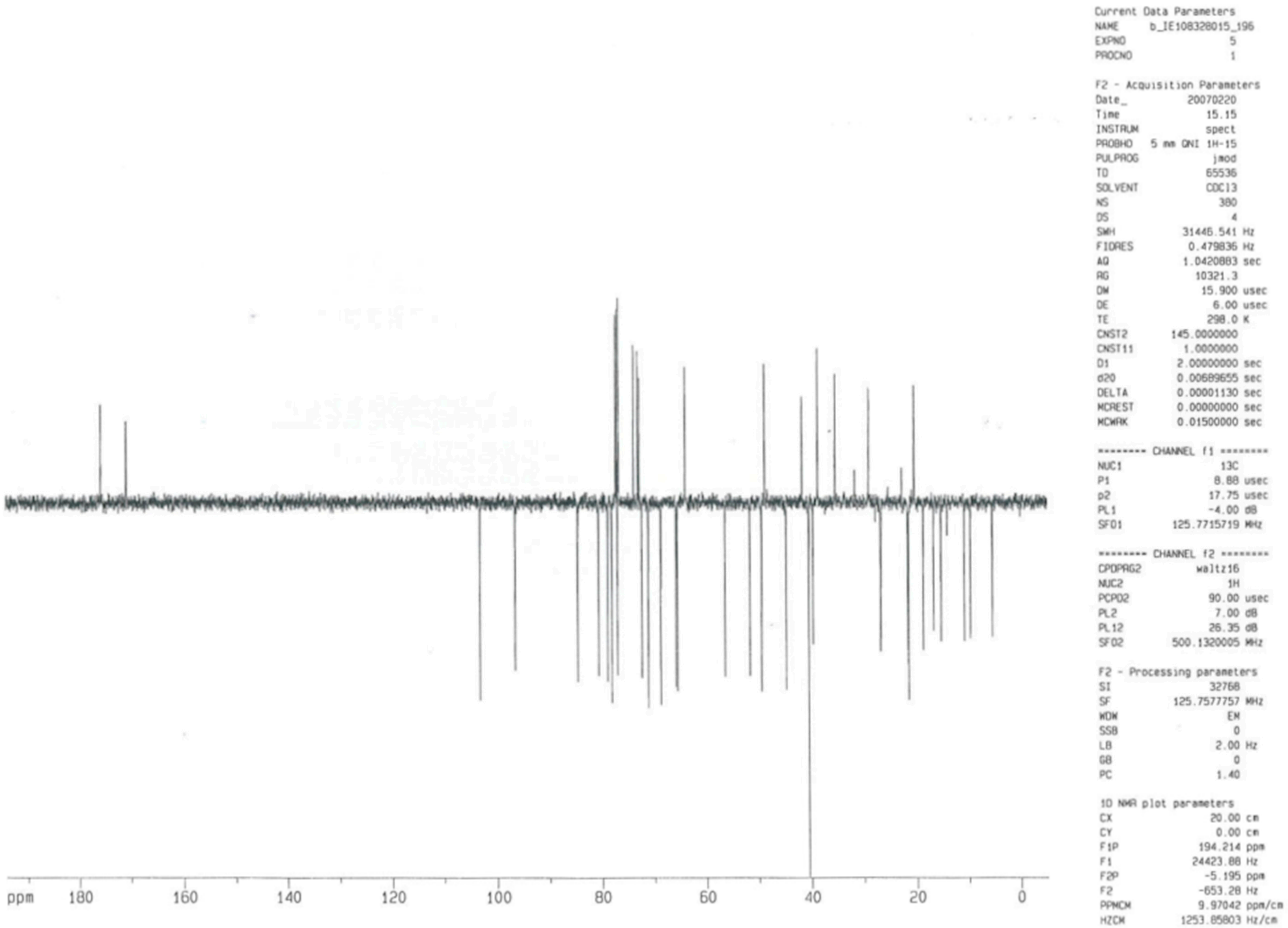

Figure S16. ^{13}*C-APT spectrum of* ***15a*** *in $CDCl_3$ at 25 °C*

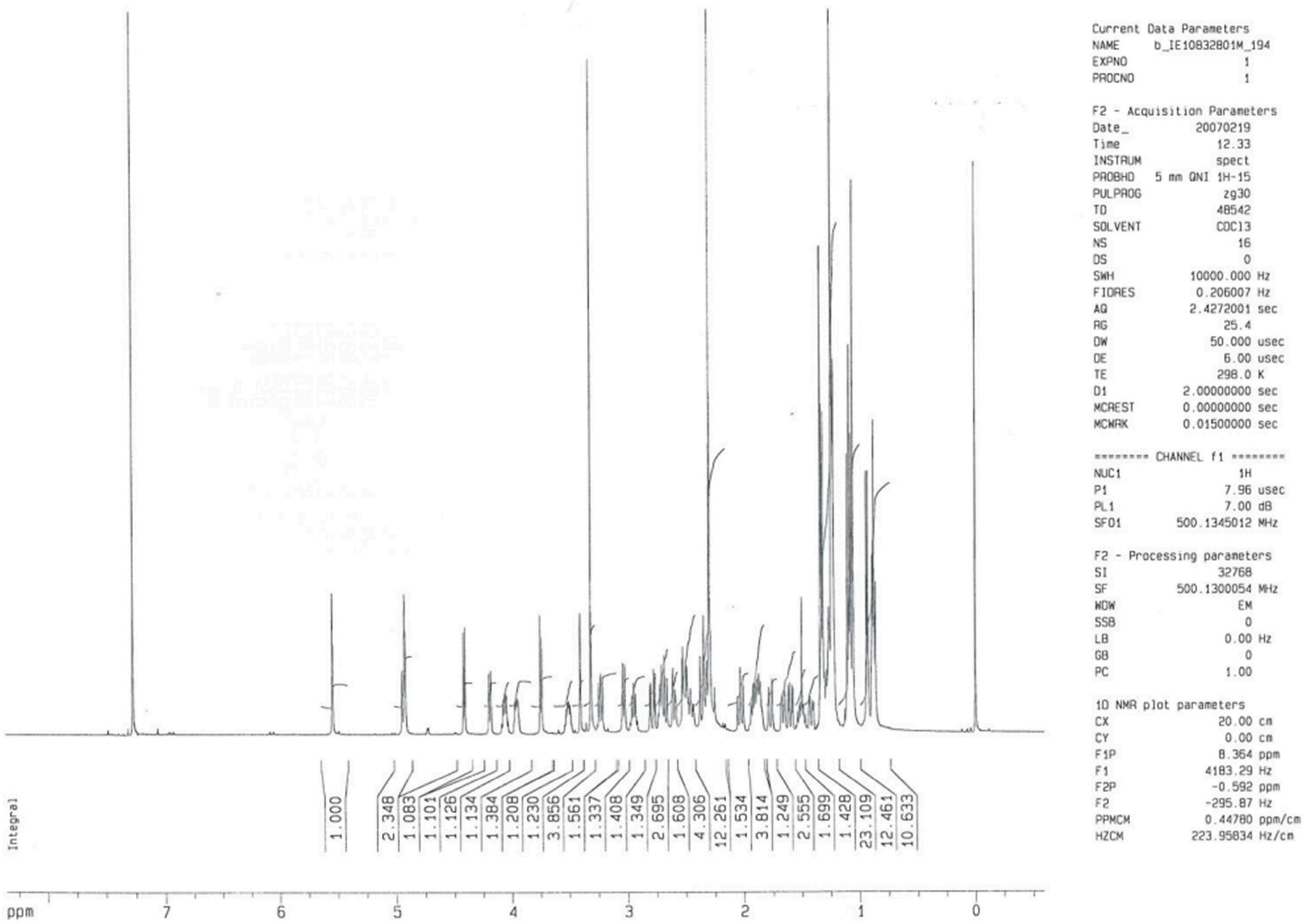

Figure S17. *^{1}H spectrum of **15b** in $CDCl_3$ at 25 °C*

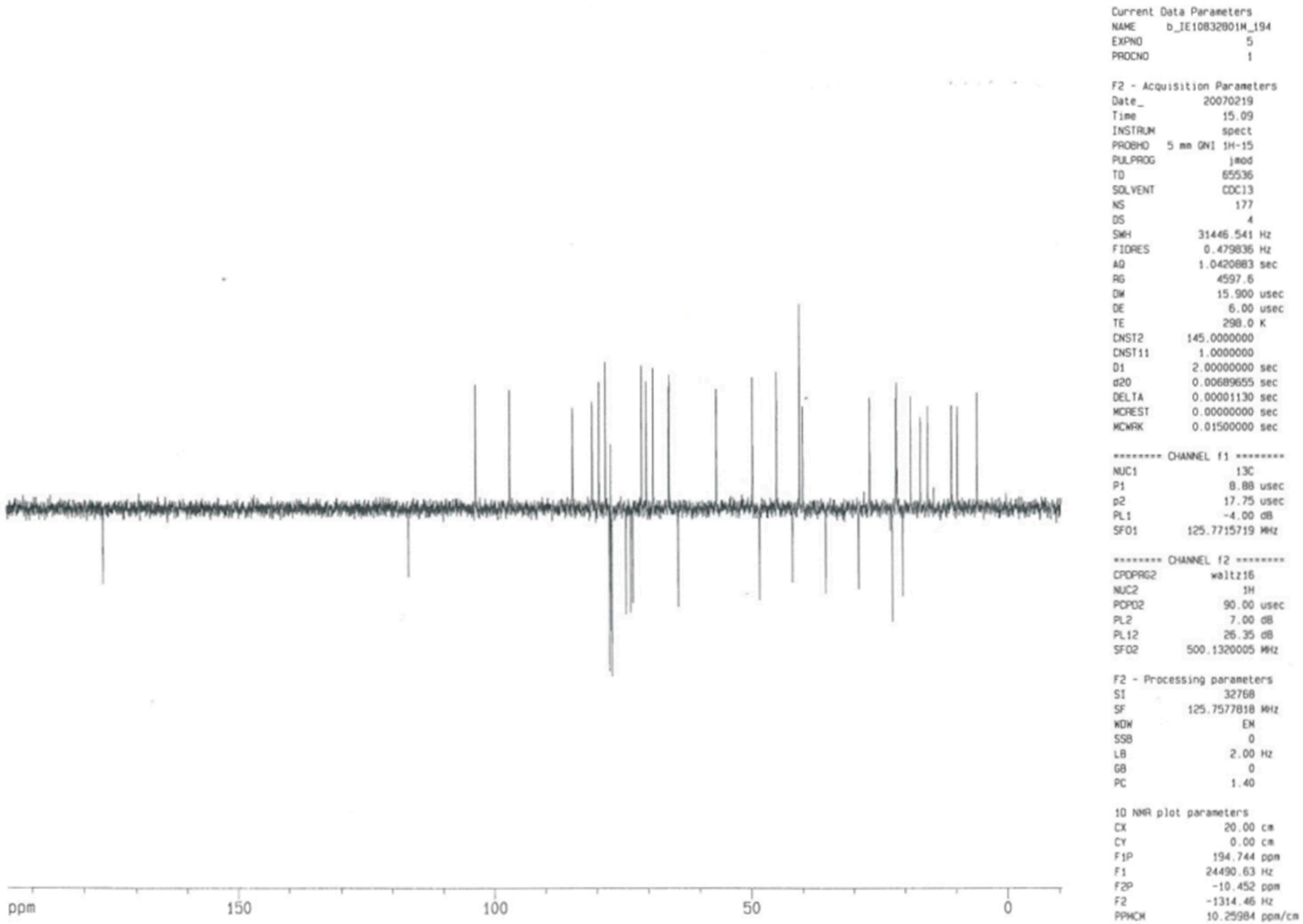

Figure S18. ^{13}C-APT spectrum of **15b** in $CDCl_3$ at 25 °C

***Table S1.** Comparison of $^{3}J_{H,H}$ coupling constants for azithromycin, **3**, **10a** and **10b** in $CDCl_3$ at 25 °C; *overlapped, the largest difference marked in red*

Atom	azithromycin	3	10a	10b	Atom	azithromycin	3	10a	10b
**2, 3**	4.0	4.1	8.7	9.0	**2, 16**	7.5	6.9	7.2	7.3
**3, 4**	2.0	1.6	1.8	1.7	**4, 17**	7.5	7.3	7.3	7.3
**4, 5**	7.5	6.7	7.0	6.1	**8, 19**	6.5	7.1	7.0	7.0
**7a, 8**	< 1	< 1	< 1	1.1	**10, 20**	6.8	6.9	6.4	6.4
**7b, 8**	*	7.4	*	*	**14, 15**	7.5	7.4	7.5	7.3
**8, 9a**	< 1	*	< 1	3.5	**5', 6'**	6.1	6.0	6.4	6.1
**8, 9b**	11.0	11.0	12.1	12.5	**5'', 6''**	6.3	6.0	6.2	6.4
**10, 11**	1.2	1.2	1.8	1.8					
**13, 14a**	2.8	1.6	2.0	2.0	**11, 11 OH**	6.6	< 1	-	-
**13, 14b**	9.8	9.4	11.1	11.1	**2', 2' OH**	+	n/a	n/a	n/a
**22, 23eq**	-	4.9	2.9	3.7	**4'', 4'' OH**	10.5	10.2	n/a	n/a
**22, 23ax**	-	7.4	10.7	10.8					
**22, L1a**	-	16.0	4.9	7.2	**7a, 7b**	14.7	14.2	14.5	14.7
**22, L1b**	-	-	8.2	4.0	**9a, 9b**	11.0	10.5	12.1	12.5
**q2, q3**	-	-		4.6	**14a, 14b**	14.4	14.5	14.4	14.4
**q3, q4**	-	-	8.5	-	**4'a, 4'b**	12.6	13.0	12.5	*
**q5, q6**	-	8.2	8.2	7.0	**2''a, 2''b**	15.2	15.0	14.7	14.7
**q6, q7**	-	7.6	6.9	8.2	**23a, 23b**	-	14.5	11.6	8.5
**q7, q8**	-	8.3	8.2	7.6	**L1a, L1b**	-	-	15.0	15.6
**1', 2'**	7.3	7.2	7.3	7.3					
**2', 3'**	10.3	9.8	10.4	9.9					
**3', 4'a**	3.8	3.6	4.0	*					
**3', 4'b**	12.2	13.0	12.0	*					
**4'a, 5'**	1.9	< 1	2.1	2.0					
**4'b, 5'**	11.0	10.4	10.2	10.2					
**1'', 2''a**	< 1	< 1	1.4	2.4					
**1'', 2''b**	5.0	4.6	4.6	4.3					
**4'', 5''**	9.6	9.6	8.8	8.8					
